# Facts and Hopes: CAR T-Cell Therapy and Immune Contexture in Non-Hodgkin Lymphoma

**DOI:** 10.1158/1078-0432.CCR-24-2267

**Published:** 2025-07-07

**Authors:** Assia Hijazi, Frederick L. Locke, Nathalie Scholler, Mike Mattie, Simone Filosto, Davide Bedognetti, Jérôme Galon

**Affiliations:** 1 INSERM, Laboratory of Integrative Cancer Immunology, Paris, France.; 2 Equipe Labellisée Ligue Contre le Cancer, Paris, France.; 3 Centre de Recherche des Cordeliers, Sorbonne Université, Université Paris Cité, Paris, France.; 4 Moffitt Cancer Center, Tampa, Florida.; 5 TORL BioTherapeutics, Culver City, California.; 6 Kite, a Gilead Company, Santa Monica, California.; 7 Veracyte, Marseille, France.

## Abstract

The genetic reprogramming of T cells with chimeric antigen receptors (CAR) specifically targeting CD19 in B-cell malignancies or B-cell maturation antigen for plasma cell tumors has achieved remarkable success. CAR T-cell therapy represents a revolutionary strategy in personalized cancer care, leveraging the immune system's precision to target cancer cells with unprecedented efficacy. However, challenges persist, with resistance and relapse occurring in hematologic malignancies. Understanding the intricate mechanisms governing response and resistance is crucial, emphasizing factors such as pharmacokinetics, product attributes, and tumor biology. This review focuses on biomarkers associated with CAR T-cell therapy in mature B-cell non-Hodgkin lymphoma malignancies, underscoring the importance of preexisting tumor immune contexture. Previous findings highlight strong correlation between early peak levels of CAR-T cells after treatment initiation and treatment response. Maintaining an optimal CAR T-cell–to–tumor burden ratio is essential for sustained responses. Systemic and tumor immune contexture affects therapy outcomes, revealing preexisting immunity's role in CAR T-cell efficacy. The mechanistic impact of CAR-T cells was investigated using pre- and posttreatment biopsies, revealing specific markers associated with treatment response in refractory large B-cell lymphoma, across patients receiving CAR T-cell therapy in the second- and third-line settings, supporting precision medicine in developing next-generation cell therapies for hematologic malignancies. The evolution of the tumor microenvironment with therapy lines was also demonstrated, supporting earlier intervention with CAR T-cell therapy. Ongoing translational efforts, including single-cell omics analysis, aim to uncover additional factors that affect outcomes to develop more potent treatments.

## Introduction

The emergence of chimeric antigen receptor (CAR) T-cell therapy marks a revolutionary advancement in cancer treatment as it involves genetically reprogramming T cells to identify and eliminate cancer cells (1, 2). CARs are synthetic receptors that integrate the binding capabilities of antibody fragments with T-cell signaling domains, effectively boosting T-cell activity against malignancies (1). These CAR-T cells can be derived from a patient’s own T cells (autologous) or from donor cells (allogeneic) and have demonstrated significant success in targeting key antigens like CD19 and B-cell maturation antigen (3), which are prevalent in B-cell malignancies and plasma cell tumors (4–6).

**Figure 1.**
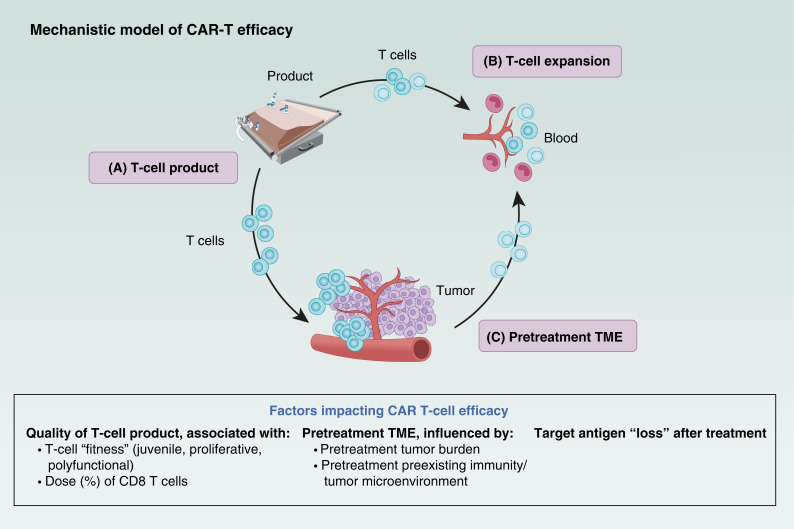
Mechanistic model of CAR-T efficacy. This cycle highlights the key factors that determine the success of CAR T-cell therapy, from the quality of the engineered T cells (T-cell product) to their expansion and interaction with the TME. **A,** T-cell product: CAR-T cells are engineered *ex vivo* to express CARs that target tumor-specific antigens. The quality of the T-cell product, including the fitness of the modified T cells (characterized by a juvenile, proliferative, and polyfunctional state), plays a crucial role in therapy success. **B,** T-cell expansion: After infusion, CAR-T cells circulate through the bloodstream, in which their ability to expand and proliferate is critical for mounting a robust immune response against the tumor. The percentage of CD8 T-cell expansion affects their therapeutic effectiveness. **C,** Pretreatment TME including factors such as pretreatment tumor burden and pretreatment preexisting immunity/TME can significantly affect CAR T-cell infiltration and activity, as well as target antigen “loss” after treatment.

CAR T-cell therapy has achieved significant clinical milestones, with seven FDA-approved products (Table 1): tisagenlecleucel (Kymriah; ref. 7), axicabtagene ciloleucel (axi-cel; Yescarta; ref. 8), brexucabtagene autoleucel (Tecartus; ref. 9), lisocabtagene maraleucel (Breyanzi; ref. 10), idecabtagene vicleucel (Abecma; ref. 11), ciltacabtagene autoleucel (Carvykti; ref. 12), and P-BCMA-ALLO1 (13). These therapies have demonstrated sustained complete remissions in subsets of patients across various B-cell malignancies (7, 8, 14), including B-cell acute lymphoblastic leukemia (B-ALL) and different types of lymphomas. Landmark trials such as ZUMA-1 [third-line (3L) large B-cell lymphoma (LBCL); refs. 8, 15] and ZUMA-7 [second-line (2L) LBCL; ref. 16] led to the approval of CAR T-cell axi-cel (Fig. 1) for treating LBCL in both lines. However, challenges remain, as a considerable proportion of patients with B-cell non-Hodgkin lymphoma (B-NHL) fail to achieve durable responses, indicating mechanisms of resistance and relapse (17).

**Table 1.** CAR T-cell products.

#	CAR T-cell therapy	Manufacturer	Indication	Systematic INN names (WHO)	Approval
1	Kymriah	Novartis	B-ALL	Tisagenlecleucel	FDA approval in 2017
2	Yescarta	Gilead Sciences/Kite Pharma	LBCL	Axicabtagene ciloleucel	FDA approval in 2017
3	Tecartus	Gilead Sciences/Kite Pharma	Mantle cell lymphoma and B-ALL	Brexucabtagene autoleucel	FDA approval in 2020
4	Breyanzi	Bristol Myers Squibb	LBCL	Lisocabtagene maraleucel	FDA approval in 2021
5	Abecma	Bristol Myers Squibb and Bluebird bio	Relapsed or refractory multiple myeloma	Idecabtagene vicleucel	FDA approval in 2021
6	Carvykti	Janssen Biotech, Inc	Relapsed or refractory multiple myeloma	Ciltacabtagene autoleucel	FDA approval in 2022
7	P-BCMA-ALLO1	Poseida Therapeutics (licensed to Roche)	Relapsed or refractory multiple myeloma	P-BCMA-ALLO1	FDA approval in 2024
8	BLA-101	Blazar Biotech	Multiple myeloma (phase I trials)	BLA-101	Clinical trial phase
9	CART-19	Tmunity Therapeutics	B-ALL (phase I trials)	CART-19	Clinical trial phase
10	SCD-101	Scinai Immunotherapeutics	Various B-cell malignancies (preclinical)	SCD-101	Clinical trial phase
11	BBT-301	Bellicum Pharmaceuticals	B-cell malignancies (preclinical)	BBT-301	Clinical trial phase
12	Regen-Cov	Regeneron Pharmaceuticals	Various cancers (phase II trials)	Regen-Cov	Clinical trial phase
13	CT053	CARsgen Therapeutics	Multiple myeloma (phase II trials)	CT053	Clinical trial phase
14	P-BCMA-201	Poseida Therapeutics	Multiple myeloma (phase II trials)	P-BCMA-201	Clinical trial phase
15	Alexis-4	Celularity	B-cell malignancies (preclinical)	Alexis-4	Clinical trial phase
16	Zylumy	Oryzon Genomics	Lymphoid malignancies (phase II trials)	Elzasonan	Clinical trial phase
17	AUTO1	Autolus Therapeutics	B-cell malignancies (phase I trials)	AUTO1	Clinical trial phase

Abbreviation: INN, International Nonproprietary Names; WHO, World Health Organization.

Identifying biomarkers linked to CAR T-cell therapy in these malignancies is crucial for optimizing treatment outcomes and addressing clinical challenges (18). Advanced techniques, such as high-throughput sequencing and multiomics analyses, allow researchers to investigate the intricate interactions between CAR-T cells and B-cell tumor microenvironments (TME; ref. 19), yielding valuable insights into biomarker discovery. Large-scale clinical trial data have revealed biomarkers that predict treatment efficacy and could aid in patient stratification (18, 20–22). For example, specific genetic markers, such as CD19 expression, significantly affect patient outcomes (18), potentially enabling clinicians to customize CAR-T therapies according to individual patient needs. This holistic approach advances personalized CAR-T therapies, improving efficacy and safety while addressing the variability among patients.

Understanding the mechanisms underlying response and resistance to CAR T-cell therapy is critical for optimizing treatment outcomes and moving CAR-T to earlier lines of therapy (23). Key factors influencing response include pharmacokinetics (PK), product characteristics, and tumor biology (8, 15). Peak levels of CAR-T cells in the blood within the initial weeks after treatment correlate strongly with response (15). Additionally, the ratio of CAR-T cells to tumor burden can play a crucial role in achieving durable responses (24). Importantly, the immune contexture of tumors and systemic immune features significantly affect the outcome to CAR T-cell therapy in LBCL (25, 26). Understanding these interactions is vital for improving treatment outcomes and developing novel therapeutic strategies.

This review focuses on biomarkers associated with outcomes in CAR T-cell therapy for B-NHL malignancies, emphasizing the importance of the preexisting tumor immune contexture.

## CAR T-Cell Expansion, Immunomodulatory Serum Analytes, and Product Features Correlate with Outcomes

Analysis of the PK data demonstrated that the number of gene-marked CAR-T cells/unit volume of blood within 2 weeks after treatment is a strong correlate of response in patients treated in the ZUMA-1 (3L) and ZUMA-7 (2L) registrational studies (8, 15, 16). This finding is concordant with earlier research employing a comparable CD19-directed CAR product, in which several blood serum analytes, including IL-15, were also associated with CAR T-cell efficacy (27). This outcome is also consistent with subsequent observations in 2L LBCL from the ZUMA-7 trial (16), in which increased IL-15 following lymphodepletion was positively associated with overall response rate (28). In addition, high baseline levels of C-reactive protein, ferritin, IL-6, and TNF-α serve as indicators of preexisting systemic inflammation, which may negatively affect treatment outcomes (29, 30).

Systemic T-cell exhaustion (26), characterized by poor lymphocyte infiltration into the TME and low levels of circulating CAR-T cells, is associated with a lack of durable response to therapy. However, a TME enriched with chemokines (CCL5 and CCL22), γ-chain receptor cytokines (IL-15, IL-7, and IL-21), and IFN-regulated molecules showed an association with T-cell infiltration and markers of activity (26).

Product T-cell fitness (Fig. 1), as determined by parameters such as doubling time during manufacturing, affects CAR-T expansion and significantly correlates with treatment response (Fig. 1; ref. 24). Specifically, T cells with shorter doubling time, indicative of better fitness, showed higher rates of expansion. Moreover, the composition of T-cell subsets within the CAR-T product, particularly less differentiated T cells, is consistently associated with improved efficacy (24, 31, 32). The differentiation status of T-cell subsets within the CAR-T product correlates with the attributes of the circulating T cells (i.e., T-cell fitness, CAR T-cell expansion, T-cell exhaustion markers, and dose of specialized T cells) collected at apheresis (24), yet such attributes are likely to also reflect the activation and differentiation status of the T cells infiltrating the tumorous lymph nodes.

Functional characteristics of CAR-T cells, measured through cytokine production in polyfunctionality assays, further contribute to treatment response (33, 34). Polyfunctional CAR-T cells, capable of engaging multiple immune programs, exhibit enhanced antitumor activity (33).

The CAR molecule design also influences treatment outcomes, for which factors like single-chain variable fragments affinity (14, 35) and signaling domains (36) significantly affect CAR-T persistence and antitumor activity (37). Optimized CAR designs, including lower-affinity single-chain variable fragments (35) and modulation of signaling pathways, aim to enhance CAR-T functionality and persistence while minimizing off-target effects (38).

Current translational efforts, including single-cell omics analysis, seek to elucidate additional factors influencing CAR T-cell therapy, including the role of the endogenous T-cell receptor repertoire, molecular mechanisms governing T-cell fitness, and the diversity of effector mechanisms (39, 40). These insights will guide the development of more potent CAR-T therapies and deepen our understanding of resistance mechanisms in cancer immunotherapy.

## The Tumor Immune Contexture Is a Correlate of Outcomes with CAR T-Cell Treatment

In solid tumors, immune contexture signatures have been identified as predictive of survival and therapeutic response, highlighting the critical role of immune factors in treatment outcomes (41). Similarly, the immune contexture of tumors is essential in assessing the clinical response to anti-CD19 CAR T-cell therapy, particularly in LBCL (26). Previous studies have shown that conventional prognostic factors and tumor characteristics, such as cytogenetic markers, molecular subtypes, and double-/triple-hit LBCL or C-MYC overexpression, do not predict outcomes with CAR T-cell therapy (26, 42). Conversely, tumor burden and TME features are strongly associated with outcomes to CAR T-cell therapy.

In-depth analysis of tumor samples from patients treated with axi-cel has revealed that the immune microenvironment before treatment significantly shapes treatment response (26). Patients with a pretreatment “hot” TME, characterized by increased density of activated CD8^+^ T cells and elevated expression of T cell–related genes, are more prone to respond to axi-cel (28, 43). Conversely, a “cold” TME with low T-cell involvement is associated with primary treatment resistance (43). However, in patients with low tumor burden, favorable CAR T-cell PK may overcome the limitations of low T-cell infiltration (44). Additionally, patients with larger tumor burdens may require both a favorable TME and high CAR T-cell expansion for tumor regression (44). Consistently, in the ZUMA-1 trial samples, clinical response and overall survival were associated with pretreatment immune contexture as characterized by Immunoscore and Immunosign 21 (IS21), which describe infiltration and activation of T cells (26). In ZUMA-1, pretreatment tumor immune contexture was associated with T-cell presence and activity in the TME through chemokines (e.g., CXCL9 and CXCL14) and cytokines (e.g., IL-15, IL-7, IL-18, and IL-21) produced locally (26), which supports the hypothesis that stromal production of T cell–attractive chemokines and γ-chain receptor cytokines may promote a T cell–involved TME generally favorable for CAR T-cell activity.

Additional pretreatment TME immune features, such as myeloid suppressive signature, are also associated with treatment outcomes. Patients with a T cell–involved microenvironment are more likely to achieve complete and durable responses, whereas those with a dysregulated myeloid-rich microenvironment may experience initial response followed by relapse (44). Moreover, systemic features such as the composition of peripheral blood immune cells before treatment also influence the TME and treatment outcomes (Fig. 1).

**Figure 2.**
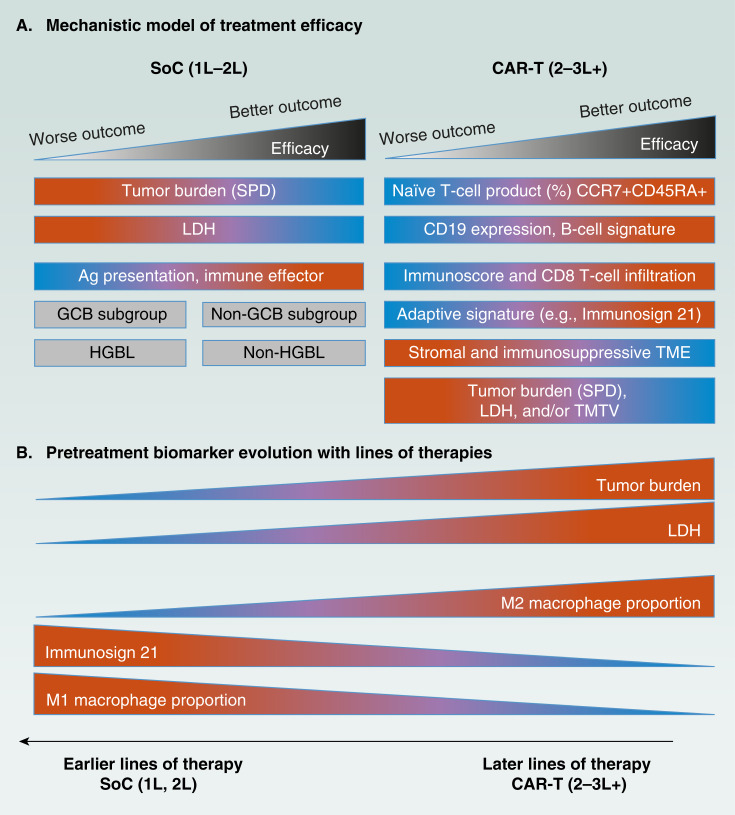
Biomarker correlates of treatment response and TME dynamics across therapy lines. **A,** Efficacy-associated biomarkers. Biomarkers associated with efficacy in SOC (1L–2L) and CAR-T (2L–3L+) arms are shown. Biomarkers are color-coded by expression levels, with red indicating higher expression and blue indicating lower expression. Tumor burden (SPD), LDH levels, and the germinal center B-cell (GCB) subtype did not influence outcomes in the CAR-T (2L–3L+) arms, whereas CD19 expression had no impact on outcomes in the SOC arm. **B,** TME biomarker evolution. Biomarker evolution across treatments highlights immune adaptation and changes in TME composition, with higher levels of LDH, tumor burden, and M2 macrophages observed in later lines of therapy, whereas IS21 and M1 macrophages decrease with lines of therapy. GCB, germinal center B-cell subtype. [Adapted from Locke and colleagues (25). http://creativecommons.org/licenses/by/4.0/.]

Following CAR T-cell therapy, the TME may evolve toward a cold immune contexture, characterized by reduced T-cell involvement and increased regulatory signature (Fig. 2A; ref. 26). This evolution may contribute to secondary resistance following CAR T-cell therapy. Patients with secondary treatment resistance exhibit increased expression of immunosuppressive myeloid genes in the TME (Fig. 2A; ref. 44).

Besides immune contexture, the target expression level on malignant cells is a strong determinant of outcomes with CAR T-cell therapy in LBCL. Pretreatment target expression levels are also predictive of response. In terms of target-related immune evasion, approximately 30% of patients with LBCL who relapsed after axi-cel therapy showed substantially lower levels of CD19 expression in their tumors (45, 46). Despite prior treatment with R-CHOP (rituximab, cyclophosphamide, hydroxydaunorubicin, oncovin, prednisone) chemotherapy, the expression of other B-cell lineage antigens such as CD20 is generally preserved. Target-related evasion occurs under the selective pressure of anti-CD19 CAR-T cells, which can lead to the emergence of antigen-negative clones from heterogeneous tumor populations (46, 47).

Future research directions include identifying genomic alterations and immune features that influence CAR T-cell outcomes, developing and validating predictive TME signatures, and exploring additional immune pathways involved in CAR T-cell therapy response. Uncovering the mechanisms of target-related immune evasion and its implications for treatment resistance is essential for improving CAR T-cell therapy efficacy (25).

Real-world evidence confirms the efficacy of CAR T-cell therapy in patients with refractory or relapsed LBCL, with consistent response rates and durable remissions observed in clinical practice (48). Key factors associated with efficacy include systemic inflammation (24), tumor burden (24, 49), and the tumor immune microenvironment (25, 26, 50, 51), which collectively contribute to treatment resistance (25, 48, 52). These findings provide valuable insights into optimizing CAR T-cell therapy for patients with LBCL.

## Mechanisms of CAR T-Cell Resistance in LBCL

Common mechanisms of resistance to axi-cel in 3L LBCL have been identified (26). Around 15% of patients experienced primary resistance, characterized by stable or progressive disease as the best clinical response (53). This resistance was associated with poor fitness of the T-cell product and limited immune involvement in the TME before treatment.

Secondary resistance, observed in patients who initially responded but later relapsed, was often linked to low expansion of CAR-T cells relative to the pretreatment tumor burden. This mechanism was more prevalent in patients with higher tumor burden and was associated with a systemic inflammatory state (24), indicated by elevated levels of myeloid-related cytokines and chemokines. Increased pretreatment myeloid markers correlated with a myeloid-rich/T cell–depleted signature in the TME (44, 54).

Some patients experienced CD19-related evasion, in which tumor cells with heterogeneous CD19 expression outgrew after CAR T cell–mediated killing of CD19-positive tumor cells (45, 46). However, death receptor signaling pathways such as fasL–fas and TRAIL–DR4/5 contribute to CAR-T efficacy and patients with high *FAS* expression receiving CAR-T therapy had significantly prolonged survival, independently of the target expression level (40). This provides evidence on the critical role of the Fas pathway in the effectiveness of T cell–based immunotherapies, emphasizing the significance of the interactions between the TME and CAR T-cell products.

Patients who achieved durable responses typically fell into two categories: those with low tumor burden and favorable product attributes, resulting in high CAR T-cell expansion, and those with high tumor burden but still achieving high CAR T-cell expansion, leading to durable complete responses (CR).

Therefore, resistance to axi-cel in LBCL can arise from various mechanisms, including poor T-cell product fitness, low CAR T-cell expansion relative to tumor burden, and CD19-related evasion. Leveraging these factors is critical for optimizing CAR T-cell therapy and improving treatment outcomes for patients with LBCL.

In LBCL, similar resistance mechanisms to axi-cel have been observed across other CAR T-cell products (55), emphasizing the importance of CAR T-cell expansion for clinical outcomes (56, 57). However, further analysis is needed, including validation in real-world settings.

**Figure 3.**
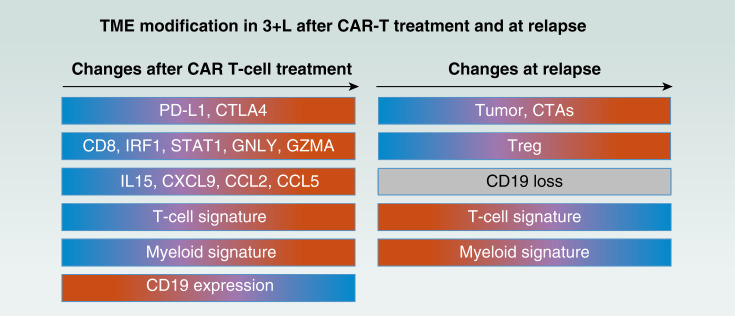
TME modification in 3L+ after CAR-T treatment and at relapse. Changes in immune cell profiles and signatures after CAR T-cell treatment are represented. Blue indicates low expression levels, red represents high expression levels, and gray reflects a complete loss of expression. A decrease in CD19 antigen expression and increase in immune cell biomarkers (PD-L1, CTLA4, CD8, IRF1, STAT1, GLNY, GZMA, IL15, CXCL9, CCL2, and CCL5), as well as in T-cell and myeloid signatures following CAR-T treatment, are shown. Changes at relapse are characterized by an increased expression of the cancer–testis antigen (CTA) expression, high levels of regulatory T cells (Tregs), and a reduction in myeloid and T-cell signatures. Complete loss of CD19 protein commonly occurs as a mechanism of relapse after CAR-T therapy. CCL2, chemokine ligand 2; CCL5, chemokine ligand 5; CD8, cluster of differentiation 8; CTLA4, cytotoxic T-lymphocyte–associated protein 4; CXCL9, C-X-C motif chemokine ligand 9; GNLY, granulysin; GZMA, granzyme A; IRF1, interferon regulatory factor 1; STAT1, signal transducer and activator of transcription 1.

Leukemias, particularly B-ALL and chronic lymphocytic leukemia, have been extensively studied with regard to response and resistance mechanisms to CAR T-cell therapy. In B-ALL, relapses after initial response often involve loss of the CD19 target, either through genetic mutations or alternate splicing variants (Fig. 3; refs. 47, 58–60). Chronic lymphocytic leukemia studies have shown an association between PK profiles, T-cell fitness, and clinical response (61), highlighting the importance of optimizing CAR configuration and manufacturing processes (62, 63).

To overcome treatment resistance, several strategies have been proposed. One approach aims to enhance the ratio of functional CAR-T cells to tumor burden within the TME through dose optimization, immune checkpoint modulation, or product engineering (64, 65). Another strategy involves broadening the immune mechanisms deployed by CAR T-cell therapy, such as through combination approaches (66) or multi-targeted CAR designs (67). Biomarker-based treatment optimization and the development of allogenic off-the-shelf CAR T-cell products are also being explored.

Machine learning analysis of data from CAR T-cell trials has identified potential actionable factors, including the pretreatment tumor and myeloid-associated inflammatory state and the percentage of infused product T cells with a juvenile phenotype (24, 26), which may guide future treatment optimizations.

Although basic mechanisms of treatment resistance related to T-cell fitness and target-related evasion are comparable across different histologies, specific elements may vary depending on the tumor type and product features. Gaining insights into these mechanisms and optimizing treatment strategies accordingly are pivotal for enhancing the effectiveness of CAR T-cell therapy across diverse cancer types.

## The Immune Contexture Predicts Response to CAR T in 2L LBCL (ZUMA-7 Trial)

ZUMA-7 is the most extensive clinical dataset available for CAR T-cell therapy in 2L LBCL (16). Our analysis of this dataset aimed to identify novel tumor biomarkers associated with key outcomes, including event-free survival (EFS), duration of response, ongoing response, CR, and objective response to CAR T-cell therapy (axi-cel; ref. 25), in comparison with the historical standard-of-care (SOC) treatment, which consists of salvage chemotherapy/high-dose therapy, followed by autologous stem cell transplant.

Translational analyses from ZUMA-7 revealed that malignant cell characteristics and TME composition influence outcomes with axi-cel and SOC differently (Fig. 2A; ref. 25). These findings provide valuable insights into the mechanisms underlying treatment response. For instance, tumor gene expression signatures representing immune contextures—including a B-cell signature and a stromal-/immunosuppressive-enriched cluster—were positively and negatively associated, respectively, with CAR T-cell therapy outcomes (25). This suggests that tumors with immunosuppressive features may reduce CAR T-cell efficacy by hindering their trafficking to malignant cells or compromising their functional state.

Notably, a cluster linked to B-cell lineage and proliferation index was positively associated with favorable clinical outcomes, particularly in patients with high-grade B-cell lymphomas (double-/triple-hit disease; ref. 25). Despite their aggressive and highly proliferative nature, these tumors may lack active cellular suppressive mechanisms, making them more responsive to CAR T-cell therapy (25).

A key finding was the distinct biomarker associations observed with axi-cel versus SOC. Although biomarkers such as the B-cell signature and CD19 protein expression were positively correlated with outcomes following axi-cel treatment (Fig. 2A), other TME immune features—including antigen processing and presentation machinery and dendritic cells—were linked to improved outcomes with SOC (25). These findings highlight the fundamental mechanistic differences between axi-cel, which relies on direct antigen engagement by CAR-T cells, and SOC, which activates endogenous immunity against tumor epitopes.

## Discussion

### Strategies to overcome treatment resistance

Despite differences in biomarker associations, outcomes with axi-cel were consistently improved compared with SOC across all presented biomarker subgroups (25). This suggests that axi-cel therapy offers advantages over SOC regardless of specific tumor characteristics. The correlation between CD19 expression on malignant cells and treatment outcomes supports recent findings by Spiegel and colleagues (68) although their study involved a much smaller dataset. Further investigation is warranted to validate these findings in the real-world clinical setting. In ZUMA-7, patients with lower CD19 expression tended to have a more complex, immune-infiltrated TME (25). Low CD19 expression was associated with TME features that negatively correlated with axi-cel EFS (20). This suggests that the relatively shorter EFS observed in these patients may be influenced not only by suboptimal target expression but also by concurrent immune contexture features. Novel gene expression signatures identified could potentially serve as predictive biomarkers for outcomes with CAR T-cell therapy (25). These TME signature clusters offer valuable insights into the mechanistic underpinnings of treatment outcomes.

Moving forward, deeper molecular characterization, including TME features and cancer cell mutational profiles, may guide treatment decisions in LBCL. Stratification based on these molecular characteristics, rather than traditional classifications, could optimize treatment selection and improve patient outcomes. However, further research is needed to fully understand the predictive value of these TME signatures and their implications for guiding therapeutic interventions in LBCL.

### Emerging biomarkers for CAR T-cell therapy management

This study identified a significant shift in key biomarkers, particularly those related to tumor immune contexture, across different lines of therapy, supporting the rationale for earlier CAR T-cell intervention. Additionally, the impact of prior treatments on CAR T-cell product fitness (28) may contribute to the varying landscape of predictive markers across treatment lines (Figs. 1 and 2B).

Product-related markers (Fig. 1A and B): Patients with a diminished B-cell signature and a less favorable tumor immune microenvironment generally experienced poorer clinical outcomes. This raises the question of whether specific CAR T-cell product characteristics could mitigate these unfavorable conditions. The findings suggest that a CAR-T product enriched in the CCR7^+^CD45RA^+^ T-cell phenotype may improve outcomes in patients with lower CD19 protein expression and heightened immunosuppressive features (25).

Tumor-associated markers (Fig. 1C): Tumors that had undergone fewer prior treatments exhibited higher levels of IS21 (Fig. 2B), a gene expression signature previously linked to enhanced immune infiltration and improved response to axi-cel (26). This may reflect changes in the TME over successive treatment lines and/or selection biases in patient survival. Additionally, as treatment lines increased, the proportion of M1 macrophages in the TME decreased, whereas M2 macrophages became more prevalent (64).

Findings from the recent ZUMA-7 study further demonstrated that axi-cel significantly outperformed SOC, particularly in patients with high tumor burden, as measured by the sum of the products of diameters (SPD) of up to six target lesions. High tumor burden, often linked to elevated lactate dehydrogenase (LDH) levels, is typically associated with aggressive disease and poor prognosis (24, 69, 70).

Interestingly, in the ZUMA-7 trial, no correlation was observed between SPD or LDH and treatment response in the axi-cel arm—contrasting with findings from 3L LBCL in ZUMA-1. Although differences in the median SPD and LDH between ZUMA-7 and ZUMA-1 may explain these discrepancies, ZUMA-7 included many patients with substantial tumor burden and elevated LDH, with a comparable SPD range with ZUMA-1 (25). This suggests that the absence of an association between SPD and outcomes in ZUMA-7 could, in part, be attributed to a more favorable TME immune contexture in the 2L setting. Furthermore, SPD and LDH may not be the most informative prognostic indicators of tumor burden, highlighting the potential value of alternative metrics such as total metabolic tumor volume (25, 26, 71).

### Genetic and immune contexture markers

Optimized tumor characteristics and T-cell fitness may enhance the therapeutic potential of first-line therapy. This is supported by recent ZUMA-12 study findings, which demonstrated a 78% CR rate and an 89% overall response rate in first-line patients with high-risk LBCL treated with axi-cel (72).

**Figure 4.**
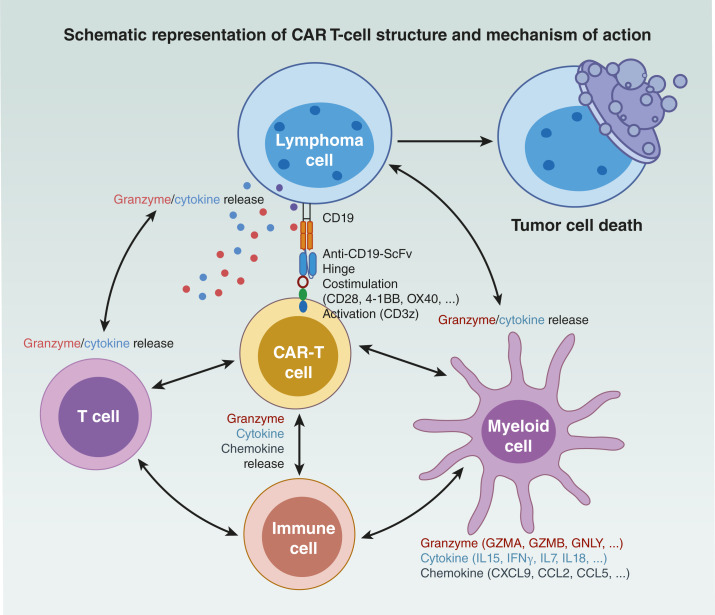
Schematic representation of CAR T-cell structure and mechanism of action. Designed CAR receptors comprising a receptor domain, CD28 co-stimulatory domain, and CD3ζ activation domain bind to CD19 antigens on the surface of lymphoma cells. This binding event initiates T-cell activation, triggering the release of granzymes and cytokines. Preexisting immune cells, including T cells and myeloid cells, support CAR-T cells through the production granzymes, cytokines, and chemokines. This process induces the targeted death of B-cell lymphoma. CCL2, chemokine ligand 2; CCL5, chemokine ligand 5; CXCL9, C-X-C motif chemokine ligand 9; GNLY, granulysin; GZMA, granzyme A; GZMB, granzyme B; ScFv, single-chain variable fragment.

Understanding the immune contexture is crucial for comprehending mechanisms of action and the likelihood of sustained response to CAR T-cell therapy (Fig. 4). In addition to SPD, metabolic tumor volume, LDH, and CD19 expression, measurements of tumor immune contexture using parameters like Immunoscore, IS21, B-cell signatures, and stromal and immunosuppressive gene signatures are emerging as important determinants of durable responses to axi-cel intervention (25). The study supports earlier intervention with CAR T-cell therapy because of a more conducive immune contexture for increased axi-cel activity, contributing to its superior efficacy compared with SOC in 2L LBCL across various prognostic subgroups. Moreover, patients with high-grade B-cell lymphoma/double-/triple-hit disease, which is associated with poorer outcomes to conventional chemoimmunotherapy, significantly benefited from axi-cel treatment (25). The enrichment of B-cell proliferation and CD19 expression in these tumors, along with fewer immunosuppressive cells, underscores the sensitivity of this high-risk population to CD19-directed CAR T-cell therapy compared with SOC. The ZUMA-12 trial showcases the remarkable efficacy of precision medicine in CAR-T therapy for high-risk LBCL (72). Evaluating axi-cel as a first-line treatment yielded impressive response rates. This success underscores the personalized approach of CAR-T therapy, marked by robust CAR-T-cell expansion in all patients (72), representing a substantial leap forward in personalized cancer care.

Overall, these observations may inform studies on patient management based on tumor biology/biomarkers and the design of next-generation therapeutics.

A limitation of these clinical trials could be the selection bias from inclusion criteria, which may not fully reflect real-world patient diversity. However, the strong outcomes observed highlight axi-cel potential for high efficacy in patients with favorable biomarker profiles. These results can guide risk stratification, maximizing benefits and helping refine CAR T-cell clinical practice.

## Conclusions and Future Directions

The data presented above offer insights into the potential mechanisms of action and resistance to autologous CAR T-cell therapy, primarily focusing on axi-cel in B-cell lymphoma, for which extensive investigation has been conducted because of the larger patient cohort. Improving our understanding and application of this knowledge is crucial for maximizing the curative potential of this innovative treatment approach. Although CAR T-cell therapy represents a groundbreaking alternative for patients with certain relapsed/refractory B-cell malignancies, there is potential for even greater benefit by introducing this treatment earlier in the disease course, possibly in the first-line setting for high-risk patients. This approach capitalizes on the more favorable tumor characteristics and enhanced T-cell fitness, without prior exposure to chemotherapy. Advancing predictive algorithms to identify patients at high risk for relapse with standard therapies and those most likely to benefit from CAR T-cell intervention is essential for this progress.

Our findings revealed a correlation between CD19 expression on malignant cells and patient treatment outcomes. Further validation using real-world CD19 testing is still required. Additionally, patients with lower CD19 expression had a more complex and immune-infiltrated TME, which may contribute to shorter EFS. This suggests that both reduced target expression and immune contexture influence therapy outcomes.

Looking ahead, several research avenues hold transformative potential for cellular therapy and immunotherapy as a whole. Beside CAR-T therapy (73), patients may also benefit from different oncology treatment (74–91). First, the development of off-the-shelf genetically engineered immune cells have shown promising preliminary outcomes, including treatment with CAR-NK cells, also revealing the critical importance of donor selection for allogeneic cell therapies (92). Second, there is a need to extend CAR T-cell therapy to other tumor types, particularly solid tumors that present greater immune mechanistic challenges. Lastly, platforms enabling safe and effective *in situ* engineering of the immune system hold promise for treating a broad range of diseases beyond oncology (93), including regenerative medicine, inflammatory and autoimmune disorders, and infectious diseases. These directions represent exciting opportunities for advancing the field of cellular therapy and revolutionizing the treatment landscape across various medical domains.

Additionally, exosomes released by CAR-T cells provide significant advantages in tumor therapy because of their strong antitumor activity and excellent biocompatibility (94). These nanoscale vesicles carry key components, such as CARs, granzyme, and perforin, which help kill tumor cells and improve targeting, especially when activated by tumor cells (95). They also contain biomolecules like miRNAs and proteins that regulate immune responses and inhibit tumor growth. Unlike live CAR-T cells, exosomes can navigate the TME without triggering cytokine release syndrome, making them effective carriers for additional therapies, such as chemotherapeutic drugs (94), enhancing their efficacy against deep-seated tumors. Innovative engineering strategies, including the modification of CARs and the loading of RNAi molecules, may correct mutations associated with tumor recurrence and improve targeting precision and effectiveness. However, challenges related to exosomes’ high production costs, storage issues, and heterogeneity must be addressed to enable their successful translation into clinical settings. Despite these hurdles, CAR T cell–derived exosomes hold great promise for advancing cancer therapies.

In conclusion, although CAR T-cell therapy has revolutionized cancer treatment, further research is needed to overcome challenges related to treatment resistance and relapse. By unraveling the intricate mechanisms governing CAR-T response and resistance, we can optimize therapeutic strategies and improve outcomes for patients with refractory hematologic malignancies.
